# STAFFING LEVELS IN OHIO’S NURSING HOMES, ASSISTED LIVINGS, AND MEMORY CARE UNITS: SHIFT VARIATION

**DOI:** 10.1093/geroni/igae098.2802

**Published:** 2024-12-31

**Authors:** Matt Nelson, John Bowblis, Heather Menne, Amy Elliot

**Affiliations:** Miami University, Oxford, Ohio, United States; Miami University, Oxford, Ohio, United States; Miami University, Oxford, Ohio, United States; Miami University, Oxford, Ohio, United States

## Abstract

In a nursing home (NH) or assisted living (AL), residents require assistance with activities of daily living and many of these residents also have cognitive impairments associated with dementia. As a result, many NHs and ALs have created dedicated memory care units (MCUs). This paper contributes to the literature on staffing in NHs and ALs by examining staffing levels for licensed nurses and other direct care staff for the overall facility and the 1 in 4 facilities that have MCUs on different shifts: day, evening, night. Data for this study come from the 2021 Ohio Biennial Survey of Long-Term Care Facilities. Staffing levels by shift were calculated as resident to staff ratios, which is the number of residents compared to number of staff by shift. Findings show that both NHs and ALs have their lowest resident-to-staff ratios, and hence most staff on the day shift (16.0 and 24.2 for licensed nurses and 10.4 and 14.2 for direct care staff, respectively). In both settings, the night shift staffing had the highest staffing ratios. Comparing the overall NH staffing to AL staffing, NHs have more staff per resident. However, on the MCU, NHs and ALs had more similar staffing levels. This suggests that NHs and ALs staff MCUs in a similar manner, but outside of the MCU, nursing homes staff better than ALs. The evidence can be used for decision making about future staffing policies.

